# Supplementary material for: Scoping review of molecular biomarkers associated with fatigue, stress, and depression in stroke survivors: A protocol
Source: PLoS One. 2023 Feb 3;18(2):e0281238. doi: 10.1371/journal.pone.0281238 (PMC9897583; doi:10.1371/journal.pone.0281238)
Supplement: S1 File — (PDF) [file pone.0281238.s002.pdf]

UNIVERSITY OF SOUTHERN QUEENSLAND

The Vice-Chancellor has great pleasure in awarding the

Research Training Program (RTP) Stipend Scholarship

To

Tarynn Potter

Professor Geraldine Mackenzie
Vice-Chancellor

CRICOS: QLD 00244B, NSW 02225M | TEQSA: PRV12081
