# Supplementary material for: Scoping review of molecular biomarkers associated with fatigue, stress, and depression in stroke survivors: A protocol
Source: PLoS One. 2023 Feb 3;18(2):e0281238. doi: 10.1371/journal.pone.0281238 (PMC9897583; doi:10.1371/journal.pone.0281238)
Supplement: Attachment — Submitted filename: Response to Reviewers1.docx [file pone.0281238.s003.docx]

Tarynn Potter

University of Southern Queensland

West Street, Toowoomba

Queensland, Australia

29^th^ November 2022

Dear reviewers,

Thank you for your considered feedback for the manuscript titled “A scoping review of molecular biomarkers associated with fatigue, stress, and depression in stroke survivors: a protocol”. The proposed adjustments have been made to the manuscript and a detailed response to feedback provided below.

Reviewer #1 Feedback:

1. *Suggest calling this a “scoping review” rather than a “systematic scoping review”.* Title has been adjusted from “systematic scoping review” to “scoping review”.
2. *Please change “Unfortunately, this is yet to translate to” to “ Unfortunately, this has yet to translate”.* The sentence on line 103 has been corrected.
3. *Previous systematic review on the same topic published in 2016 and a few other reviews since then. What therefore is the rationale for the present review? The prior studies were not referenced by the authors*. Lines 75-77 have been updated to better reflect the previous reviews that have been conducted into biomarkers for post-stroke depression. We have also provided a more robust rationale with edits to lines 88-90 to better clarify the knowledge gap the review aims to address.
4. *The current thinking is that depression likely runs an entire clinical spectrum from mild to severe; there are genetic and neurobiological studies lending support to the notion that these conditions are not discrete categories but rather have common biological underpinnings and may form at least part of a continuum or affective disorder spectrum. This should at least be briefly mentioned as it has important implications for diagnosis and research and would further strengthen the need for precise biomarkers.* Thank you for flagging this for consideration. This reference has been included and the implications to diagnostic and therapeutic processes outlined on lines 100-103.

Reviewer #2 feedback:

1. *A major problem that repeats across the entire manuscript is the inconsistency in and interchangeable use of major descriptors of the protocol including fatigue, anxiety, stress, depression, sleeplessness, and quality of life*. Thank you for flagging the inconsistencies with these descriptors. The protocol has been reviewed and the major descriptors updated to reduce these inconsistencies. This includes updates to line 16 to provide more consistency as well as consideration for clear articulation of the main variables considered in this protocol in the substantial edits for lines 49-61.
2. *Abstract: Line 23: What these several gaps that remain?* Lines 22-24 have been updated to include detail of the current gaps in understanding.
3. *Introduction: Lines 45-62: Too many repetitions of facts. These paragraphs can be shortened into in short and succinct paragraph*. This section has been updated from lines 47-61 to concisely articulate key concepts with greater clarity.
4. *Line 93: Consider replacing "thorough understanding" with "review".* Line 112 has been updated with this change.
5. *Methods and Analysis: Lines 125-126: How will the understanding lead to increase in specific diagnostic and therapeutic interventions to guide future research? This sentence is confusing and misleading*. This statement has been removed (lines 144-146) as it did not add value to the rationale of this paragraph.

Thank you for your consideration of this protocol for publication, and for the time taken to provide your feedback to the authors. The protocol has been updated in response to reviewer feedback and should present a more concise, thorough representation of the benefits this review will contribute to the current understanding of molecular biomarkers associated with stroke comorbidities. If you have any further questions or concerns, please do not hesitate to contact me.

Kind regards

Tarynn Potter
